# Antitussive and Anti-inflammatory Dual-active Agents Developed from Natural Product Lead Compound 1-Methylhydantoin

**DOI:** 10.3390/molecules24132355

**Published:** 2019-06-26

**Authors:** Yang Xu, Fang Wang, Hongye Guo, Shihan Wang, Shuling Ni, Yan Zhou, Zhihan Wang, Huiwei Bao, Yongsheng Wang

**Affiliations:** 1 School of Pharmaceutical Sciences, Jilin University, Changchun, Jilin 130021, China; 2 College of Basic Medical Sciences, Jilin University, Changchun, Jilin 130021, China; 3 Department of Drug and Food Sciences, Changchun Medical College, Changchun, Jilin 130031, China; 4 College of Chinese Medicine Materials, Jilin Agricultural University, Changchun 130118, China; 5 Department of Physical Sciences, Eastern New Mexico University, Portales, NM 88130, USA; 6 College of Pharmacy, Changchun University of Chinese Medicine, Changchun, Jilin 130117, China

**Keywords:** *Oviductus Ranae*, natural product, 1-methylhydantoin, dual-active, antitussive, anti-inflammatory, acute pneumonia

## Abstract

Natural products play an important role in drug discovery. This work employed a natural product 1-methylhydantoin as the lead compound to develop novel dual-active drugs. 1-Methylhydantoin was isolated from *Oviductus Ranae*, which is a traditional Chinese medicine that has been used for tussive and inflammation treatment for a long time. An in silico study screened the more active 1-methylhydantoin derivatives. Antitussive assessment indicated that the newly synthesized agent had similar bioactivity with the natural product. An anti-inflammatory model used xylene induced ear edema model. At the same dosage (100 mg/Kg), the newly prepared agent had an inhibition rate 53.18% which was much higher than that of the lead compound (22.69%). The results might be ascribed to the cyclooxygenases-1 (COX-1) and cyclooxygenases-2 (COX-2) selectivity, and the fitness of the compound, and the binding pocket. The anti-particulate matter (PM 2.5) acute pneumonia was evaluated through an in vivo model constructed by nasal instillation with PM 2.5 suspension. The results of the above models suggested that this novel agent had remarkable antitussive, anti-inflammatory, and anti-PM 2.5 acute pneumonia activities.

## 1. Introduction

**Figure 1:**
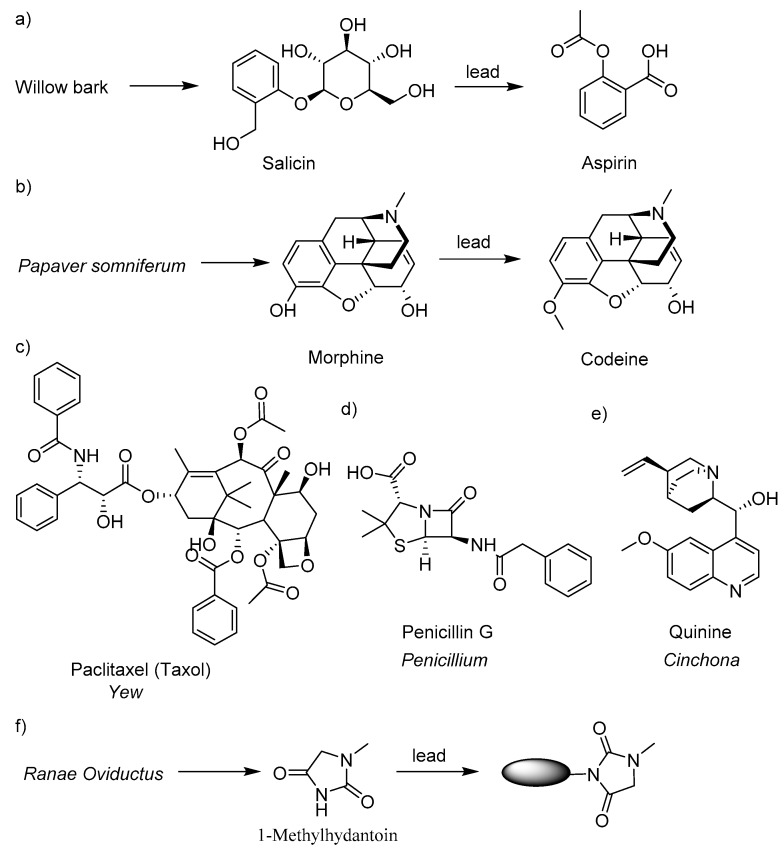
Examples used natural product (NP) resources in drug discovery. (**a**) Aspirin was developed from natural product salicin. (**b**) Codeine was prepared using morphine as the lead compound. (**c**) The anticancer drug paclitaxel was isolated from yew. (**d**) and (**e**) Penicillin G and quinine were isolated from *Penicillium* and *Cinchona* respectively. (**f**) The strategy of developing dual-active agents using a natural product lead compound 1-methylhydantoin.

Natural products (NPs) and drugs have been linked to each other for thousands of years [1,2,3]. NPs are referred to the constituents or metabolites of animals, plants, insects, marine organisms and microorganisms, as well as many endogenous chemical components in animals, which are characterized by structural diversity and easy to bind to biological macromolecules [4,5,6,7]. NPs which have played a significant role in drug discovery are the main resources of innovative drugs, drug candidate structures, and drug lead structures [8,9]. More than 50% of modern drugs were developed from NPs and their derivatives [10]. Figure 1a–e presents several well-known drugs which were developed from NPs or NP derivatives. For example, aspirin (ASP) is a natural product derivative from willow bark [11]. The antitussive drug codeine was prepared using morphine, a natural product isolated from *Papaver somniferum*, as the lead compound [12]. Paclitaxel (PTX) was isolated from Pacific yew in 1971 and used for the treatment of lung cancer, breast cancer, ovarian cancer, cervical cancer, and pancreatic cancer [13,14,15]. Penicillin, the first antibiotic drug, was isolated from the mold *Penicillium*. The anti-malarial drug quinine was first isolated from the bark of a *cinchona* tree [16]. Currently, the isolation of biologically active compounds from natural products has become a popular interest in the development of new drugs. Using the active ingredients isolated from natural medicines as the lead compounds can greatly improve the hit rate of newly designed molecules, contract the developing cycle, and avoid the high development cost of chemical synthetic drugs [17,18,19]. 

*Oviductus Ranae* is a traditional Chinese medicine that has a long history being used as an antitussive, anti-inflammatory, anti-fatigue, immunity enhancing, and antilipemics drug [20,21,22,23,24]. Due to the bioactivities and high nutrition, *Oviductus Ranae* has been reputed as “natural health soft gold” [22,25]. 1-Methylhydantoin (1-MHD) was isolated from *Oviductus Ranae* (Figure 1f). Studies showed that 1-MHD demonstrated antitussive and anti-inflammatory bioactivities [26,27]. In our previous work, a series of hydantoin derivatives were synthesized to investigate the antitussive and anti-inflammatory bioactivities [27]. The results indicated that 1-MHD is a promising antitussive and anti-inflammatory drug. 

Using a natural product as the lead compound can reduce the drug developing time and increase the hit rate [28]. This work was started from the molecular design using 1-MHD as the lead compound. The inhibition of cyclooxygenases (COXs) played an important role in reducing inflammation, because COXs can catalyze the conversion of arachidonic acid to different prostaglandins (PGs) such as PGG_2_ and PGH_2_ which will induce inflammation [29,30]. Interestingly, in silico study indicated that 1-MHD had similar inhibitions in both of COX-1 and COX-2 which might be attributed to the molecular size of 1-MHD. The 1-MHD has the molecular size that can fit both COX-1 and COX-2 binding pockets. The expression of COX-1 in normal tissues promotes the synthesis of physiologically required PGs and plays an essential role in maintaining the stability of the intracellular environment and protecting the integrity of the gastrointestinal mucosa [31,32]. When COX-1 is inhibited, the gastrointestinal mucosa loses its protective effect and causes adverse reactions such as the gastrointestinal tract [31,32,33]. As an inducible enzyme, COX-2 is mainly expressed in inflammatory cells [34,35]. COX-2 can cause the level increasing of PGs in the inflammation site and promote inflammatory reaction and tissue damage [33,34,35]. Hence, the anti-inflammatory effect mainly comes from the inhibition of COX-2 [35,36]. This work demonstrated a series of agents which have the potential of selective inhibition of COX-2. On the other hand, 1-MHD has high water solubility, which may hinder the passage of 1-MHD through the cell membrane. Hydrophilicity and hydrophobicity are reported to be very important in the biological activity of a drug [37,38]. If 1-MHD is attached to a hydrophobic group, the biological activity of 1-MHD may increase. Inspired by drug combination, we used ibuprofen, aspirin, indomethacin, and naproxen as the hydrophobic moieties. Herein, we synthesized a series of derivatives of 1-MHD and investigated the bioactivities based on the results of the in silico study.

## 2. Result and Discussion

### 2.1. Molecular Design and Synthesis

**Figure 2:**
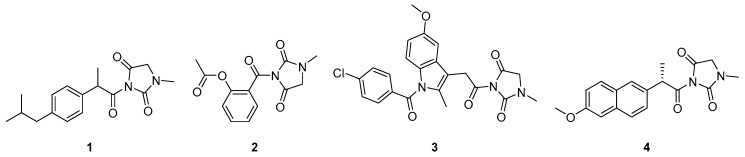
Optimization of lead compound 1-methylhydantoin (1-MHD). The hydrophobic moieties were selected from nonsteroidal anti-inflammatory drugs. The designed 1-MHD derivatives were ibuprofen conjugated 1-MHD (**1**), aspirin conjugated 1-MHD (**2**), indomethacin conjugated 1-MHD (**3**), and naproxen conjugated 1-MHD (**4**).

The optimization of the lead compound 1-MHD was focused on the imide nitrogen atom (Figure 1f). The molecular structures of designed potential antitussive and anti-inflammatory dual-active agents are presented in Figure 2. Because of the electron withdrawing groups locating adjacently to the nitrogen atom and the lone pair delocalization of the nitrogen atom, the nucleophilicity of the imide is weak. To solve this issue, the carboxylic acid groups on the acid moieties have to be converted to acyl chloride. Compared to the carboxylic acid derivatives, acyl chloride is the most reactive form. The electronegativity of chlorine is higher than carbon and oxygen. This property causes the electrons to be pulled toward the chlorine and the carbonyl carbon to be more electrophilic. Because chloride is an excellent leaving group, weak nucleophiles can attack the carbonyl group. Once the acid chloride is obtained, a nucleophilic substitution reaction takes place between 1-MHD and the acid chloride in a high yield.

**Figure 3:**
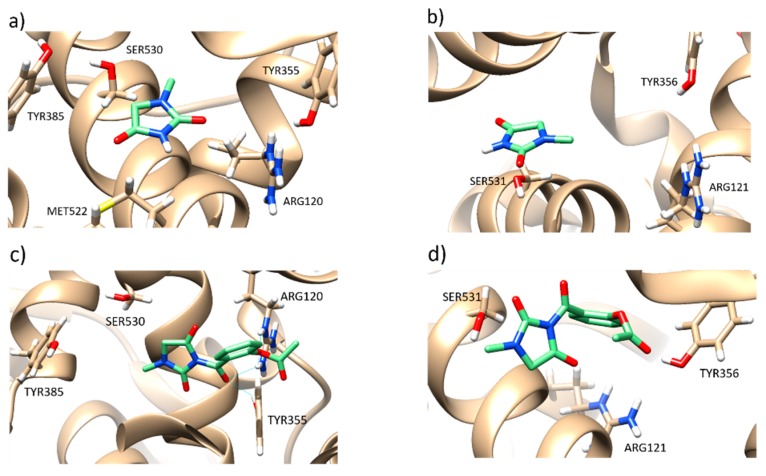
(**a**) and (**b**) Binding model of 1-MHD with cyclooxygenases-1 (COX-1) and cyclooxygenases-2 (COX-2). (**c**) and (**d**) Binding model of **2** with COX-1 and COX-2.

In silico study revealed that 1-MHD had no significant difference in the inhibition of COX-1 and COX-2 (Figure 3a,b) while **2** showed high selectivity between COX-1 and COX-2 (Figure 3c,d). The binding affinity toward the target COX-1 and COX-2 were –5.8 and –8.0 kcal/mol, respectively. ASP can inhibit COX-1 by irreversible acetylation of Ser-530, whereas 1-MHD moiety in **2** occupies the binding region of ASP and Ser-530 [30,39,40]. In contrast, **2** showed better inhibition in the COX-2 binding pocket. The docking results indicated that compound **3** was not suitable for the binding pockets in both COX-1 and COX-2 because of the molecular size. Compounds **1** and **4** have similar binding affinities but are not as good as compound **2**. According to the in silico study, this work focused on the evaluation of compound **2**. The use of an in silico approach to design bioactive agents may help reduce the drug developing period.

**Figure 4:**
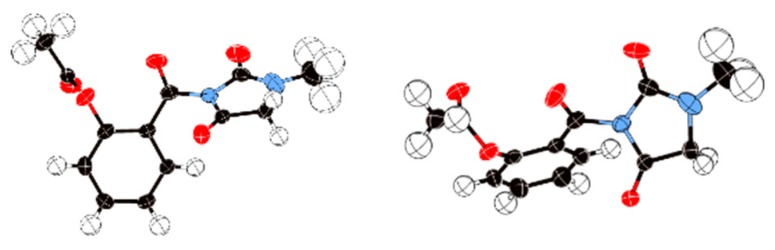
X-ray single crystal analysis. Left: Chemical structure of **2**. Middle: Top view of the X-ray single crystal structure. Right: Side view. Oak Ridge thermal ellipsoid plot program (ORTEP) for crystal structure represents 50% possibility.

Compound **2** was synthesized using the method described above. The single crystals of **2** were prepared using a 200 mg sample dissolved into 20 mL of ethyl acetate solvent. The X-ray single crystal structure of **2** possessed a monoclinic crystal system with P2_1_/_n_ space group. The Z value of the crystal was 4. Figure 4 shows the top view and side views of the X-ray single crystal structure of **2**.

### 2.2. Bioactivity Evaluation

**Figure 5:**
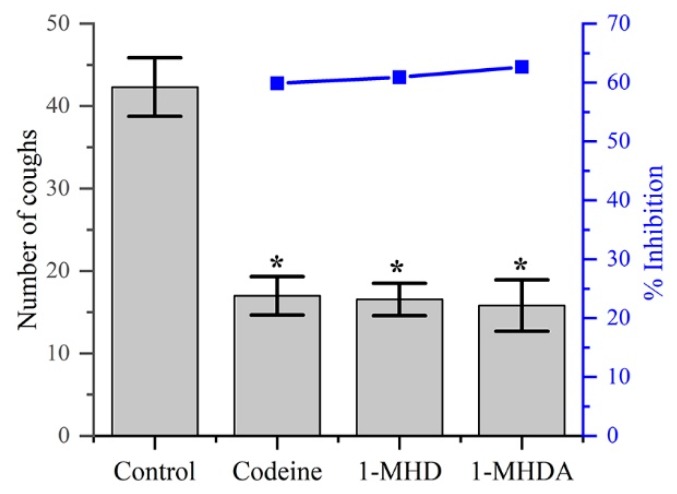
The antitussive plots of number of coughs and the inhibition percentages of different agents.

As shown in Table 1 and Figure 5, **2** obviously reduced the number of coughs in the ammonia-induced cough model compared to the control group. On the other hand, there was no significant difference in the frequency of coughs between the groups treated with codeine phosphate (30 mg/Kg), 1-MHD, and **2** (100 mg/Kg). The inhibition rate of groups with treatments of codeine phosphate, 1-MHD, and **2** appeared to be between 59 to 62%.

**Figure 6:**
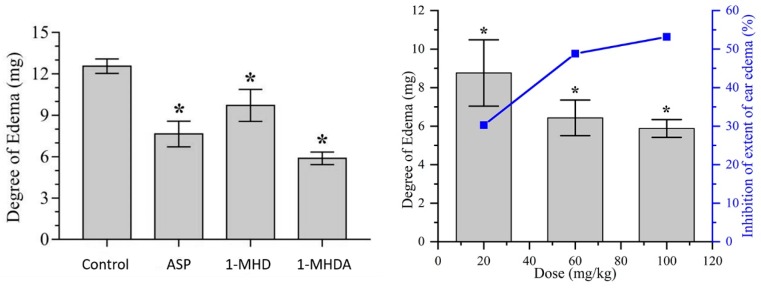
(**a**) The efficacy of different agents at the same dosage 100 mg/Kg. (**b**) the inhibition of **2** at different dosage level. * *p* < 0.05, for comparison to treated groups with control.

The anti-inflammatory activity was evaluated through xylene induced ear edema in vivo model. The anterior and posterior surfaces of the right ear of the mouse were induced using 30 μL/ear of xylene. The change in body weight caused by xylene is shown in Table 2. From Table 2, we can easily see that the 1-MHD 100 mg/Kg group had relatively low anti-inflammatory activity compared to the ASP 100 mg/Kg group but still exhibited decent anti-inflammatory activity (inhibition rate 22.69%). This might be caused by the high aqueous solubility of 1-MHD. On the contrary, **2** with 100 mg/Kg showed a salient inhibition rate 53.18%. Figure 6a visually demonstrates the differences between the control group and the experimental groups with the same dosage. The **2** group was assessed with different dose levels as well. The low dose **2** group had a higher inhibition rate than 1-MHD. This result may be attributed to the increased hydrophobicity which can lead to easy cell membrane passage. The doses evaluation of **2** showed that as the dose of **2** increased, the inhibition rate of inflammation increased. Figure 6b shows that as the dose increased, the inhibition rate tended to be smooth. This study suggests that the newly developed **2** demonstrated significant anti-inflammatory activity.

**Figure 7:**
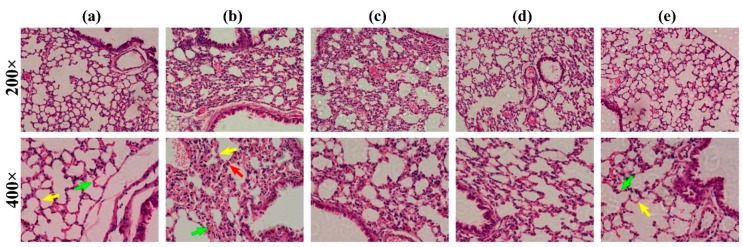
Lung tissue histology analysis. (**a**) Representative hematoxylin and eosin-stained mouse lung sections with control group. (**b**) Mouse lung sections of PM 2.5 group. Arrows point out the thickened alveolar septum, dilated telangiectasia, and neutrophil. (**c**), (**d**), and (**e**) Mouse lung sections with treatment of 1-MHD, ASP, and **2**, respectively. Note: Red arrow, neutrophil; yellow arrow, alveolar septum; green arrow, capillaries.

In this study, the lung injury induced by PM 2.5 particles and the protective effects of the drug groups were confirmed. Typically, the lung tissue consists of alveolar and alveolar septum where the alveolar wall is a thin layer of connective tissue with dense capillaries and abundant elastic fibers, and the alveolar septum is covered with flat alveolar cells. As illustrated in Figure 7a, no inflammatory cells such as neutrophils and lymphocytes were infiltrated in the control group. In contrast, thickened alveolar septum, dilated telangiectasia, and massive neutrophil infiltrations were observed in the PM 2.5 group (Figure 7b). The lead compound 1-MHD showed thinner alveolar septum, inconspicuous telangiectasia, and less neutrophils compared with the PM 2.5 group (Figure 7c). The alveolar septum and capillaries of the **2** group were close to the control group (Figure 7a,e). In addition, the lung tissue section in Figure 7e showed a significant reduction of neutrophils in the **2** group. From Figure 7c–e, we can clearly see that the PM 2.5 treatment of **2** was the most prominent and much better than the treatment of lead compound 1-MHD. 

## 3. Materials and Methods

### 3.1. Reagents and Instruments

**2** was synthesized in our laboratory. The purity of **2** was ≥ 99% by HPLC analysis. HPLC grade methanol and acetonitrile were purchased from Xinkeao Scientific and Technology Co. Ltd. (Beijing, China). FTIR spectrum was carried out on IRPrestige-21, SHIMADZU. MS was recorded by LCMS-8050, SHIMADZU. Other chemicals and reagents were of analytical grade and commercially available. All chemicals and reagents were used without further purification unless otherwise stated. The reaction was monitored by thin layer chromatography (TLC) using a silica gel coated glass plate (Qingdao Ocean Chemical Co., Qingdao, China) and visualized under UV light (254 nm). ^1^H-NMR and ^13^C-NMR were recorded on a Bruker AVANCE 500 NMR spectrometer (Fällanden, Switzerland) and chemical shifts were reported in ppm. X-ray single crystal diffraction was obtained by using a Bruker Kappa Apex II Duo single crystal diffractometer. Melting point was recorded on an SGW^®^ X-4 microscope melting apparatus (Shanghai Scientific and Technology Co. Ltd., Shanghai, China). NMR, FTIR, UV/Vis, and MS spectra of prepared compounds, single crystal data of **2** are given in Supplementary Material. 

### 3.2. Synthesis of 3-(2-(4-isobutylphenyl)propanoyl)-1-methylimidazolidine-2,4-dione (1)

The synthesis of 1-methylhydantoin-ibuprofen has been described in our previous work [27]. ^1^H-NMR (CDCl_3_, 400 MHz) *δ*: 0.89 (d, 6H, 2 × CH_3_), 1.49 (d, 3H, CH_3_), 1.83‒1.85 (m, 1H, CH), 2.42 (d, 2H, CH_2_), 2.93 (s, 3H, CH_3_), 3.77 (s, 2H, CH_2_), 4.85 (q, 1H, CH), 7.07 (d, 2H, 2 × ArH), 7.16 (d, 2H, 2 × ArH); ^13^C-NMR (CDCl_3_, 100 MHz) *δ*: 172.58, 166.93, 152.86, 140.88, 136.68, 129.57, 129.57, 127.64, 127.64, 50.85, 46.62, 44.97, 30.06, 29.52, 22.32, 22.32, 18.57. 

### 3.3. Synthesis of (2-(3-methyl-2,5-dioxoimidazolidine-1-carbonyl)phenyl acetate) (2)

Aspirin (1.0 g, 5.6 mmol) was added to a 50 mL of three-necked round bottom flask with thionyl chloride (0.6 mL). One drop of N,N-dimethylformamide (DMF) was added to the flask as a catalyst. The mixture was heated to 80 °C for 2 h and then diluted with 10 mL of chloroform. Another solution was prepared by adding 1-methylhydantoin (0.8 g, 7.0 mmol) and pyridine (1.5 mL) to chloroform (10 mL). The aspirin chloride solution was added dropwise to the 1-mehtylhydantoin/pyridine solution in ice bath. Afterwards, the mixture was stirred for 10 h at room temperature. Once the reaction completed, the mixture was poured into 30 mL of water. The organic layer was separated and washed with brine (10 mL × 3) and dried over sodium sulfate. After concentration, the crude product was purified through column chromatography with ethyl acetate/hexane (5:1) to give compound **2** (1.3 g, 83.93%) as a white solid. Single crystals of **2** (C_13_H_12_N_2_O_5_) were prepared using 200 mg. A suitable crystal was selected and put on a Bruker APEX CCD area detector diffractometer. The crystal was kept at 191(2) K during data collection. The structure was solved with the ShelXS-1997 structure solution program using Direct Methods and refined with the ShelXL-1997 refinement package using CGLS minimization. M.p.: 112–113 °C. FTIR: 2941, 1799, 1760, 1739, 1699, 1604, 1581, 1488, 1450, 964 cm^−1^. MS [C_13_H_13_N_2_O_5_]^+^ 277.08 found 227.01. ^1^H-NMR (500 MHz, CDCl_3_), *δ* ppm: 2.27 (s, 3H, CH_3_), 3.00 (s, 3H, CH_3_), 3.99 (s, 2H, CH_2_), 7.19–7.30 (m, 1H, ArH), 7.32-7.35 (m, 1H, ArH), 7.57–7.62 (m, 2H, 2 × ArH). ^13^C-NMR (100 MHz, CDCl_3_), *δ* ppm: 168.70, 166.68, 163.15, 152.56, 149.35, 134.08, 130.68, 125.77, 123.54, 51.25, 29.64, 20.76.

### 3.4. Synthesis of 3-(2-(1-(4-chlorobenzoyl)-5-methoxy-2-methyl-1H-indol-3-yl)acetyl)-1-methylimidazolidine-2,4-dione (3)

A total of 0.25 mL of thionyl chloride was dissolved in 20 mL of dichloromethane and stirred at room temperature for 15 min after adding 5 drops of DMF. Then, indomethacin (1.0 g, 2.8 mmoL) was dissolved in 5 mL of dichloromethane and added to the above reaction liquid. After reacting for 3 h at room temperature, the solution was spun dry and dissolved in 10 mL of dichloromethane. Another solution was prepared by adding 1-methylhydantoin (0.35 g, 3.1 mmoL) and pyridine (1 mL) to dichloromethane (10 mL). The indomethacin chloride solution was slowly added dropwise to the 1-methylhydantoin solution under ice bath., and the reaction was carried out for 12 h at room temperature. Finally filtered, spin-dried, pyridine removed and recrystallized from ethyl acetate to give compound **3** (0.85 g, 67%) as a white solid. M.p.: 102–103 °C. FTIR: 2835, 1816, 1745, 1708, 1677, 1618, 1593, 1479, 1469, 754 cm^−1^. MS [C_23_H_20_ClN_3_O_5_ + Na]^+^ 476.3. ^1^H-NMR (400 MHz, CDCl_3_), *δ* ppm: 2.34 (s, 3H, CH_3_), 3.03 (s, 3H, CH_3_), 3.82 (s, 3H, CH_3_), 3.93 (s, 2H, CH_2_), 4.34 (s, 2H, CH_2_), 6.66‒6.68 (m, 1H, ArH), 6.91‒6.94 (m, 2H, 2 × ArH), 7.46‒7.48 (m, 2H, 2 × ArH), 7.65‒7.67 (m, 2H, 2 × ArH). ^13^C-NMR (100 MHz, CDCl_3_), *δ* ppm: 168.53, 168.30, 166.87, 156.10, 152.92, 139.31, 136.77, 133.82, 131.19, 130.80, 130.53, 129.12, 114.89, 111.94, 110.98, 101.07, 55.68, 51.10, 34.01, 29.66, 13.58.

### 3.5. Synthesis of ((S)-3-(2-(6-methoxynaphthalen-2-yl)propanoyl)-1-methylimidazolidine-2,4-dione) (4)

Naproxen (1.0 g, 4.3 mmoL) was dissolved in 30 mL of dichloromethane, 2 drops of DMF were added dropwise with stirring, and 0.9 mL of oxalyl chloride was added under ice bath. After reacting for 5 h at room temperature, the reaction solution was spun dry, and dissolved in 10 mL of dichloromethane. Then, 1-methylhydantoin (0.55 g, 4.8 mmoL) was dissolved in 2 mL of pyridine, and the naproxen chloride solution was slowly added dropwise to the 1-methylhydantoin solution under ice bath. After the dropwise addition, the reaction was carried out for 12 h at room temperature. Finally filtered, spin-dried, pyridine removed and recrystallized from methanol to give compound **4** (1.11 g, 79.12%) as a white solid. M.p.: 100.5–101.6 °C. FTIR: 2937, 1801, 1755, 1708, 1629, 1604, 1483, 1444, 1392, 788cm^−1^. MS [C_18_H_18_N_2_O_4_ + Na]^+^ 349.2. ^1^H-NMR (400MHz, CDCl_3_), *δ* ppm: 1.55 (d, 3H, CH_3_), 2.87 (s, 3H, CH_3_), 3.72(s, 2H, CH_2_), 3.90 (s, 3H,CH_3_), 5.04 (q, 1H, CH), 7.09‒7.14 (m, 2H, 2 × ArH), 7.35(d, 1H, ArH), 7.65‒7.70 (m, 3H, 3 × ArH). ^13^C-NMR (100 MHz, CDCl_3_), *δ* ppm: 172.41, 166.93, 157.72, 152.72, 134.71, 133.72, 129.29, 128.87, 127.45, 126.63, 126.37, 118.98, 105.46, 55.24, 50.74, 46.90, 29.39, 18.55.

### 3.6. Animals

ICR male experimental mice (purchased from Changchun Yisi Experimental Animal Technology Co., Ltd., Changchun, China) and male Kunming mice (purchased from the Animal Experimental Center of Changchun University of Traditional Chinese Medicine, Changchun, China) were kept under standard pathogen-free conditions (tap water, constant room temperature 20–22 °C, 55 ± 5% humidity, a 12- hour light–dark cycle, and unrestricted access to food and water). The study protocol 20190044 was approved by the Animal Experimental Ethics Committee of College of Pharmacy of Jilin University. All experimental operation was followed for the laboratory animal care principles, and all experiments were carried out in accordance with the Guide for the Care and Use of Laboratory Animals (released by the National Research Council). 

### 3.7. Antitussive Activity 

The antitussive activity was evaluated on mice using ammonia aerosol stimulation test (1). Kunming male mice (20‒22 g) were divided into four groups randomly (*n* = 10 for each group). The control group was given 5% carboxymethylcellulose-Na (CMC-Na) solution. The standard group received codeine (1.5 mg/mL) and the other two groups were treated with 1-methylhydantoin and compound **2** respectively for seven days via continuous intragastric administration, at a concentration of 4 mg/mL in 5% CMC-Na solution. One hour after the last oral administration, mice were placed in glassware filled with ammonia aerosol. The antitussive effects of the target compounds on mice were evaluated by the frequency of cough caused by ammonia and the latent period of cough after administration. The number of coughs was detected in two minutes. Some typical actions used as special concern to strictly distinguish cough in mice from sneeze. For example, the cough can be identified by opening mouth accompanying sound of coughing and contraction of thoracic and abdomen muscles [41]. The equation of percentage of inhibition of cough times was as follows:

%Inhibition = [(T_0_−T_t_)/T_0_ × 100%] (1)

where T_0_ was the cough time of control group and T_t_ was the cough time of the treatment group. 

### 3.8. Anti-Inflammatory Activity 

Anti-inflammatory activity of the two target compounds was assessed using a xylene-induced mouse ear edema model. Kunming male mice (20‒22 g) were divided into six groups randomly (*n* = 10 for each group). One group was kept as the control group and the others were given test compounds including one group given aspirin as the standard group. The test compounds were suspended in 5% carboxymethylcellulose-Na respectively, and different doses were administered intragastrically for seven consecutive days. One hour after the last oral administration, 0.02 mL xylene was applied to the right ear of each mouse. One hour after xylene application, mice were sacrificed by cervical vertebra dislocation and a portion of ear (8 mm of diameter) was punched out and weighed. The ear weights of each group were recorded for the evaluation of anti-inflammation effect [42]. The anti-inflammatory effects were assessed by the weight difference using the following equation:

S = (W_r_ − W_l_) (2)

where W_r_ was the weight of right ear and W_l_ was the weight of left ear. The equation for percentage of inhibition of extent of ear edema was as follows:

%Inhibition = [(S_0_−S_t_)/S_0_ × 100%] (3)

where S_0_ was the extent of ear edema of control group and St was the extent of ear edema of the treatment group.

### 3.9. Anti-PM 2.5 Acute Pneumonia Bioactivity

The atmospheric PM 2.5 particles were continuously collected by HY-100WS high-load particle sampler at the roof of Tiebei Xintai Primary School, Changchun, Jilin, China (about 10 m high, with no obvious pollution source around). The atmospheric PM 2.5 particles was removed to a sealed circular box using a sterile brush to sweep the PM 2.5 particulate materials and stored in a low temperature dry environment. The experimental animals were randomly divided into five groups: Control group, PM 2.5 group, 1-methylhydantoin group, aspirin group, and compound **2** group. The acute pneumonia model was constructed giving a suspension of 40 mg/Kg of PM 2.5 to the mice through nasal installation [43]. Then, the mice were given a 0.5% CMC-Na suspension of 20 mg/Kg once per day for three days through intragastric administration. The mice were sacrificed 1 h after the administration on the third day. The lungs were removed, and the mice were perfused with a fixative solution containing 4% Formalin in phosphate buffered saline (PBS). The lunges were rinsed with 4% neutral Formalin with 0.1 M PBS (PH = 7.0) through the trachea for 2 h. The trachea was ligated. The lungs were immersed in 4% buffered Formalin for 48 h. After alcohol dehydration, the lung tissues were embedded in paraffin and stained with hematoxylin and eosin (HE). The tissues were cut into 3–4 µm sections. The slides were evaluated using optical microscope.

### 3.10. Statistical Analysis

All of the data were analyzed by on-way analysis of variance (ANOVA) and presented as the mean ± S.D. The variations were evaluated using Graph Pad Prism 7 software (Version 7.0, GraphPad Software Inc., San Diego, CA, USA). A *p* < 0.05 was considered to show significant differences. 

### 3.11. In Silico Study

Crystal structure was obtained from protein data bank (PDB entry 3n8z, Sidhu et al. [44]) with resolution 2.9 Å and (PDB entry 4ph9, Orlando et al. [45]) 1.81 Å using X-ray diffraction. Before the docking process, the crystal structure was first processed adding polar hydrogens via AutoDock Tools. Afterwards, water molecules and ligand were deleted from the binding site. Since COX-1 and COX-2 are homodimers, both the docking receptors used chain A. The structure of **2** was minimized. Grid parameters were x (−20), y (50), z (10) with box size 40 × 40 × 40. The in silico study was carried out via AutoDock Vina. 

## 4. Conclusions

In summary, this work developed a dual-active agent **2**. The bioactivities of the newly developed agent were evaluated using in vivo models. The antitussive activity indicated the newly developed agent **2** had similar antitussive activity with the lead compound. The anti-inflammatory test showed that the newly developed drug 2 has enhanced anti-inflammatory activity. The results of anti-PM 2.5 model were similar to the anti-inflammatory test which showed a significant improvement in **2** compared to the lead compound. This enhanced inflammatory effect may be due to the increased selectivity. This work initiated a study of dual-active drugs for 1-MHD derivatives.

## Figures and Tables

**Table 1:** Effects of different drug on the ammonia liquor induced cough in mice.

Group	Dose (mg/Kg)	No. of Animals	Latent Period Cough (s)	No. of Coughs	Inhibition (%)
Control	5% CMC	10	27.20 ± 5.73	42.30 ± 3.55	-
Codeine phosphate	30	10	51.50 ± 5.41*	16.98 ± 2.33*	59.86
1-MHD	100	10	38.20 ± 9.69*	16.54 ± 1.96*	60.90
2	100	10	41.00 ± 4.25*	15.80 ± 3.12*	62.65

Values expressed as mean ± SD. (n = 10), * *p* < 0.05, for comparison to treated groups with control.

**Table 2:** Effects of different drug on the xylene induced ear edema in mice.

Group	Dose (mg/Kg)	No. of Animals	Degree of Edema (mg)	Inhibition (%)
Control	5% CMC	10	12.56 ± 0.52	-
ASP	100	10	7.65 ± 0.93*	39.09
1-MHD	100	10	9.71 ± 1.16*	22.69
2	100 (high dose)	10	5.88 ± 0.46*	53.18
2	60 (medium dose)	10	6.43 ± 0.92*	48.81
2	20 (low dose)	10	8.76 ± 1.72*	30.25

Values expressed as mean ± SD. (n = 10), * *p* < 0.05, for comparison to treated groups with control.

## Supplementary Materials

The following are available online at https://www.mdpi.com/1420-3049/24/13/2355/s1: NMR, FTIR, UV/Vis, and MS spectra of prepared compounds, single crystal data of **2**.
