# Supplementary material for: Antitussive and Anti-inflammatory Dual-active Agents Developed from Natural Product Lead Compound 1-Methylhydantoin
Source: Molecules. 2019 Jun 26;24(13):2355. doi: 10.3390/molecules24132355 (PMC6651114; doi:10.3390/molecules24132355)
Supplement: Supplementary file 1 [file molecules-24-02355-s001.pdf]

Antitussive and Anti-inflammatory Dual-active Agents Developed from Natural Product Lead Compound 1-Methylhydantoin

Yang Xu [1,2,3], Fang Wang [2], Hongye Guo [1], Shihan Wang [4], Shuling Ni [1], Yan Zhou [1], Zhihan Wang [5], Huiwei Bao [6] and Yongsheng Wang [1,*]

[1] School of Pharmaceutical Sciences, Jilin University, Changchun, Jilin 130021, China; xuyangbaohuiwei@163.com (Y.X.); guohy18@mails.jlu.edu.cn (H.G.); nisl17@mails.jlu.edu.cn (S.N.); zhouyan17@mails.jlu.edu.cn (Y.Z.)
[2] College of Basic Medical Sciences, Jilin University, Changchun, Jilin 130021, China; wf@jlu.edu.cn
[3] Department of Drug and Food Sciences, Changchun Medical College, Changchun, Jilin 130031, China; xuyangbaohuiwei@163.com
[4] College of Chinese Medicine Materials, Jilin Agricultural University, Changchun 130118, China; S.W. wsh8805@163.com
[5] Department of Physical Sciences, Eastern New Mexico University, Portales, NM 88130, USA; zhihan.wang@enmu.edu
[6] College of Pharmacy, Changchun University of Chinese Medicine, Changchun, Jilin 130117, China; baohuiwei@163.com
* Correspondence: mikewangwys@outlook.com or wys@jlu.edu.cn

1. NMR spectra 2
2. FT-IR Spectrum 8
3. MS Data 11
4. UV/Vis 13
5. Crystal Data 14

1. NMR spectra

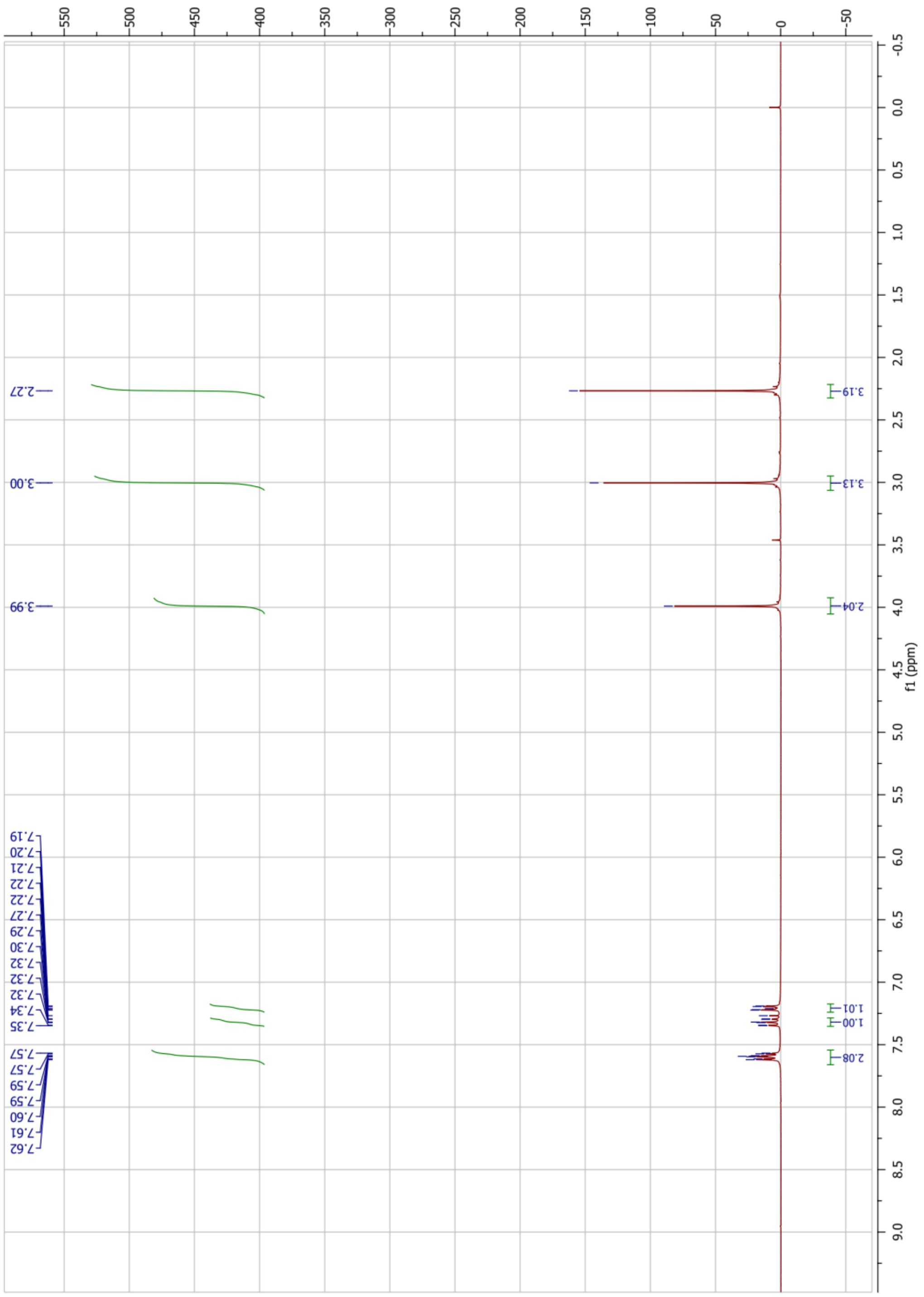

Figure S1 ^{1}H-NMR spectrum of 1-MHDA.

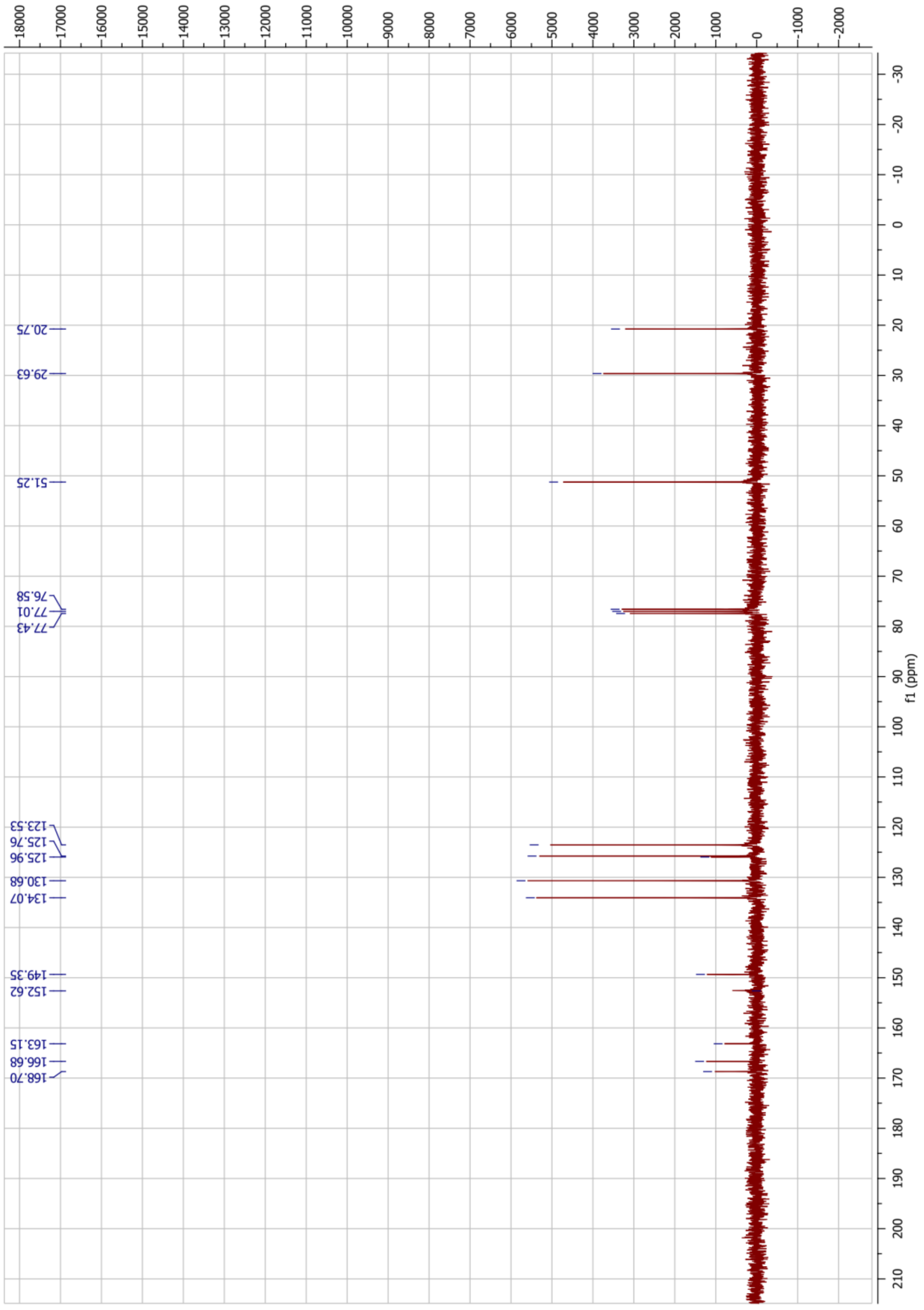

Figure S2 ^{13}C-NMR spectrum of 1-MHDA.

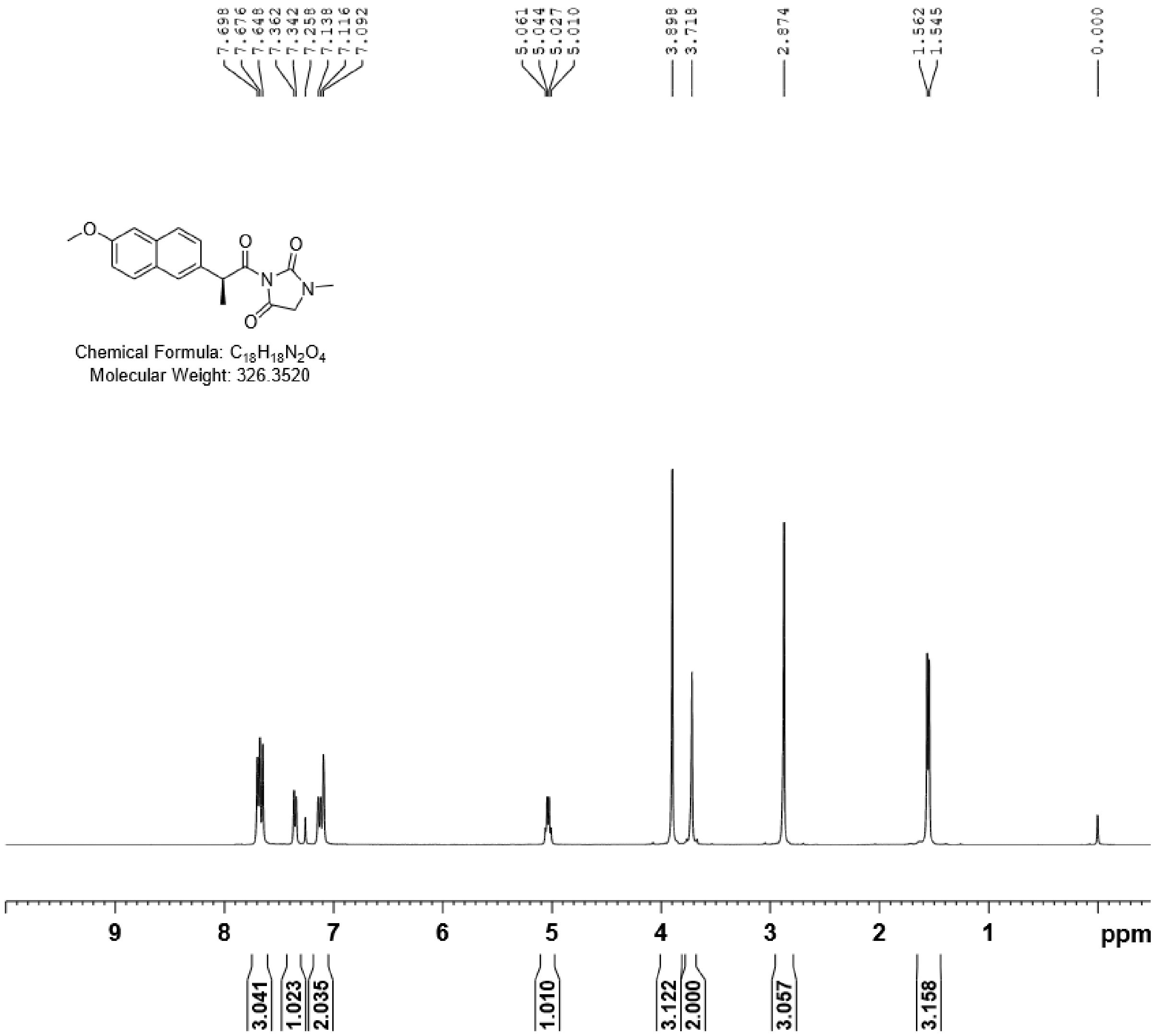

Figure S3 ^{1}H-NMR spectrum of 1-methylhydantoin-naproxen.

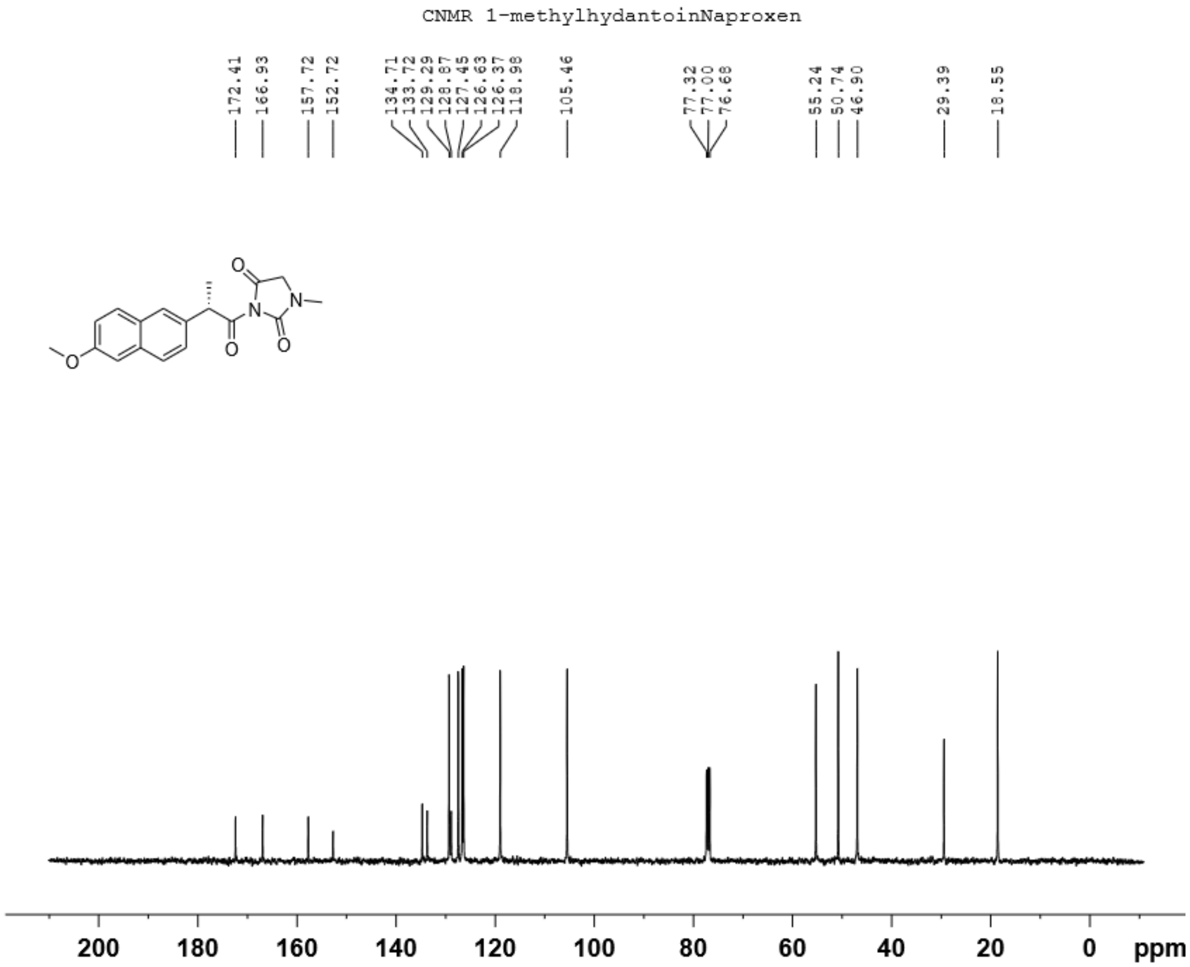

Figure S4 ^{13}C-NMR spectrum of 1-methylhydantoin-naproxen.

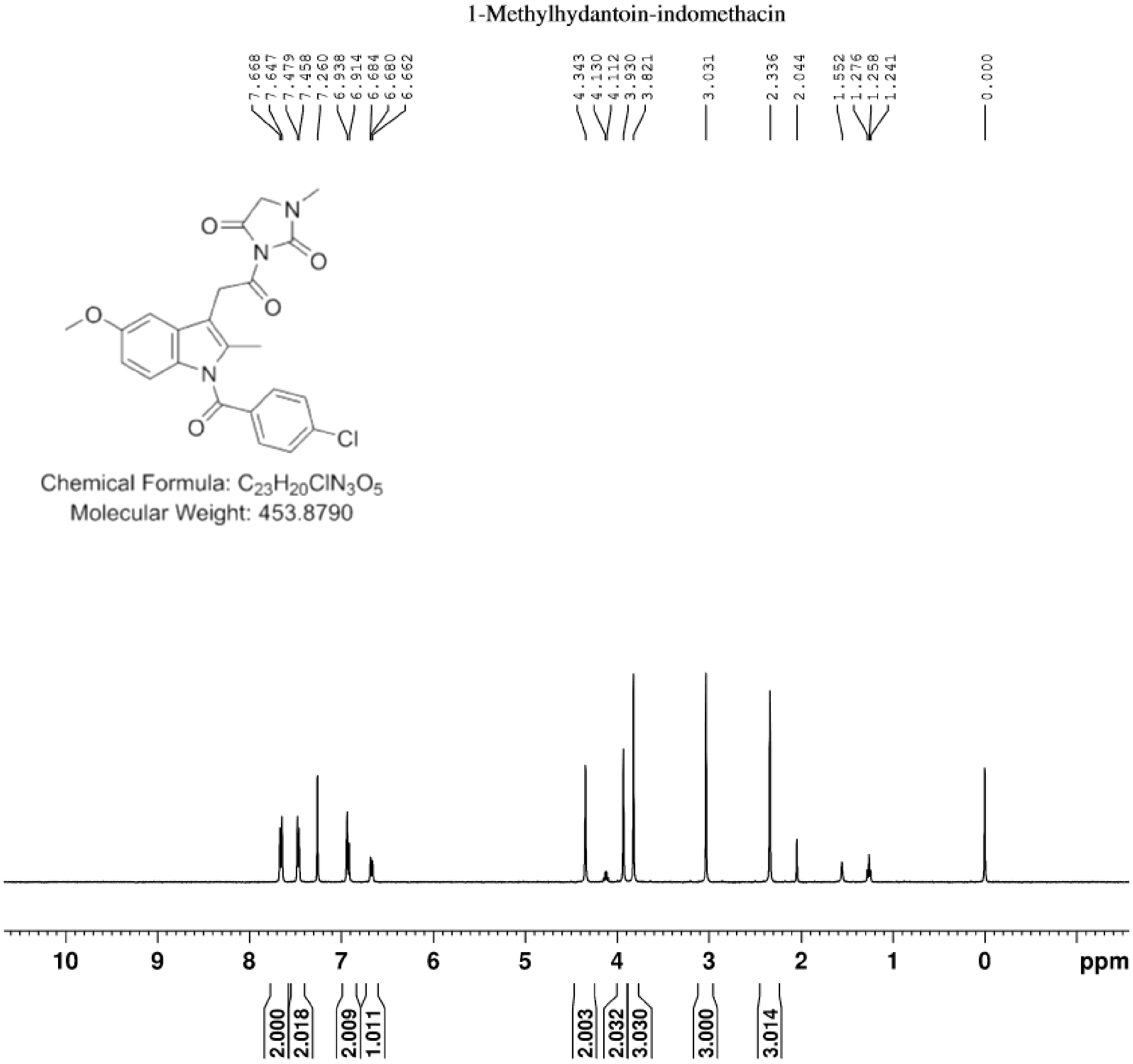

Figure S5 ^{13}C-NMR spectrum of 1-Methylhydantoin-indomethacin.

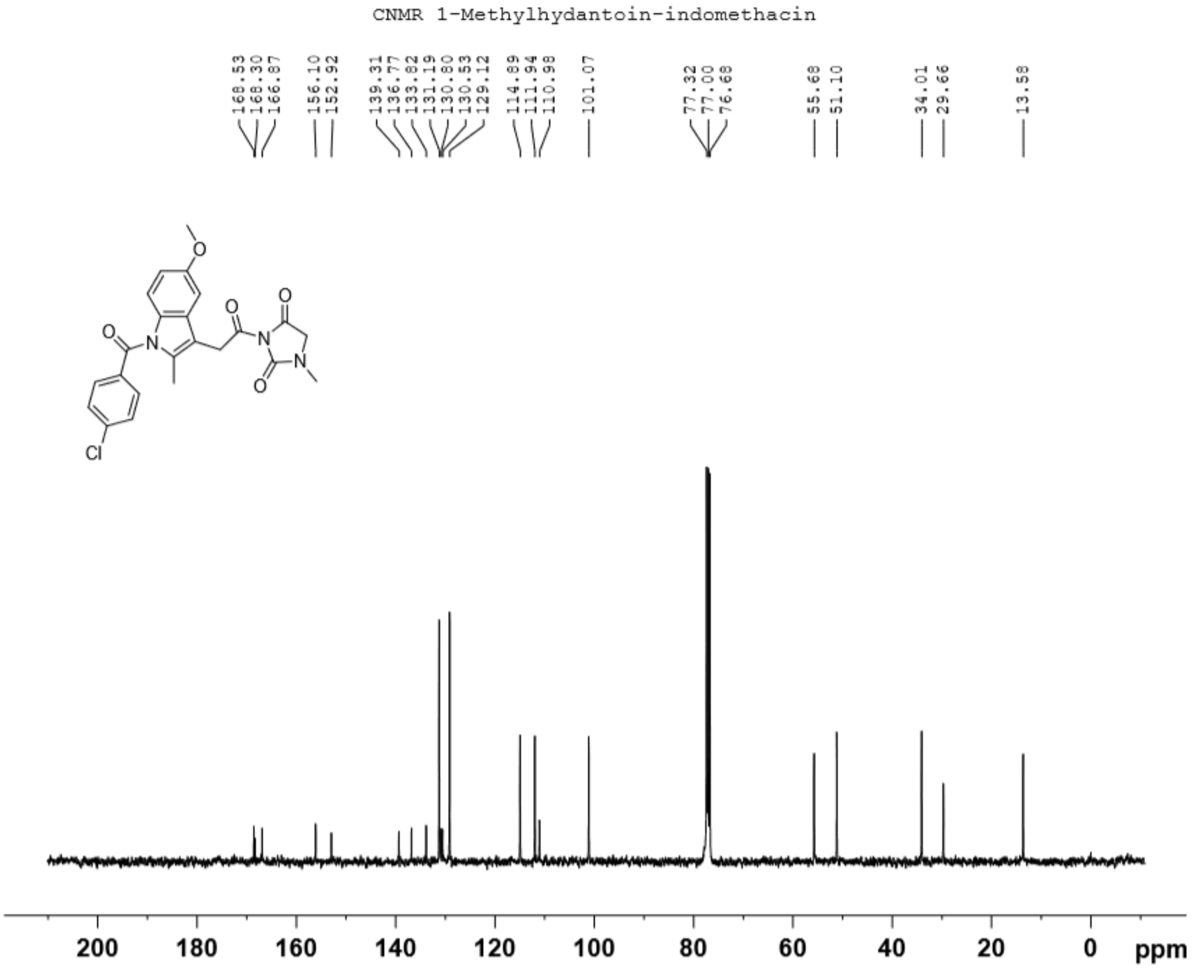

Figure S6 ^{13}C-NMR spectrum of 1-Methylhydantoin-indomethacin.

2. FT-IR Spectrum

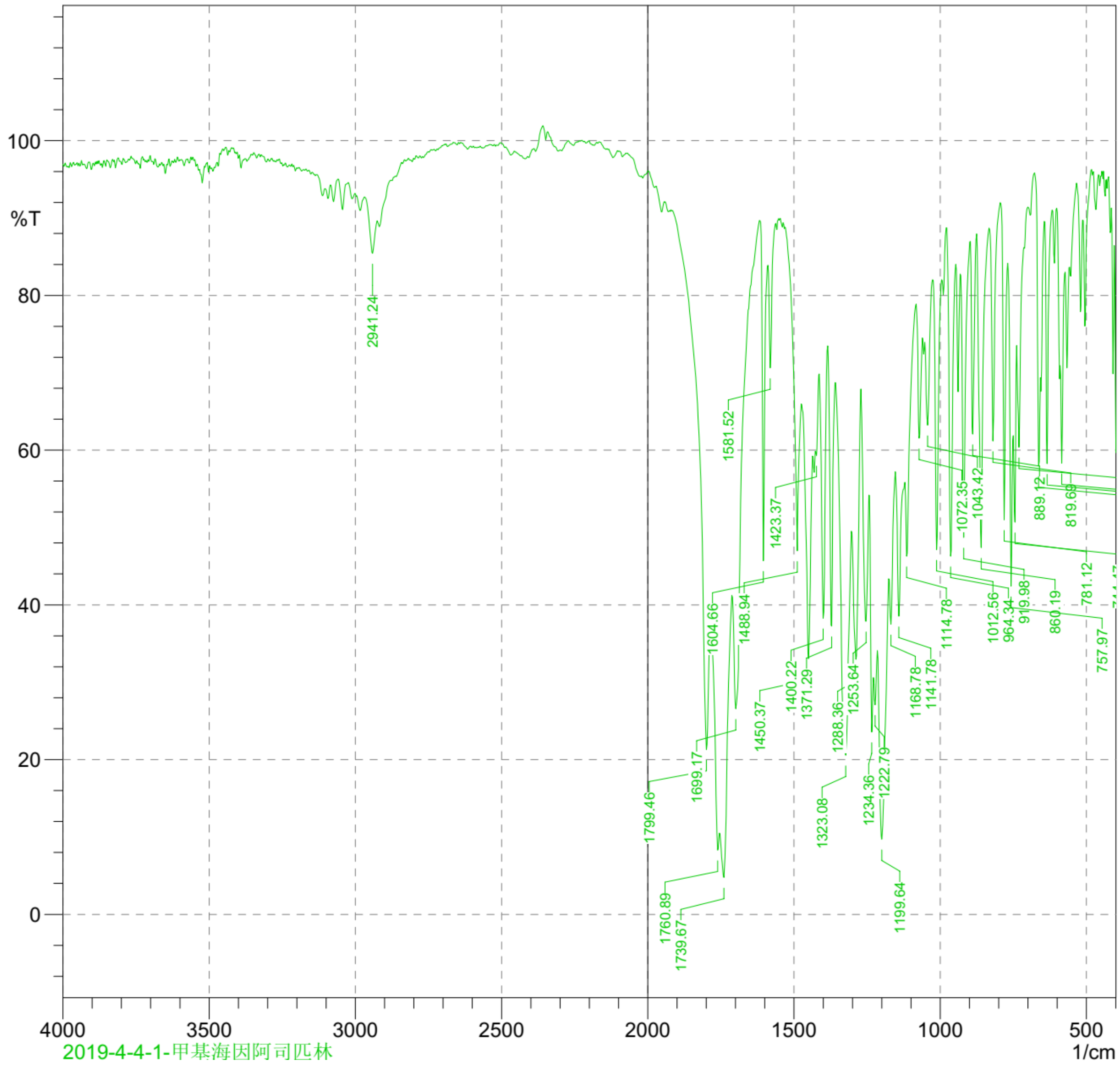

Figure S7 FT-IR spectrum of 1-MHDA.

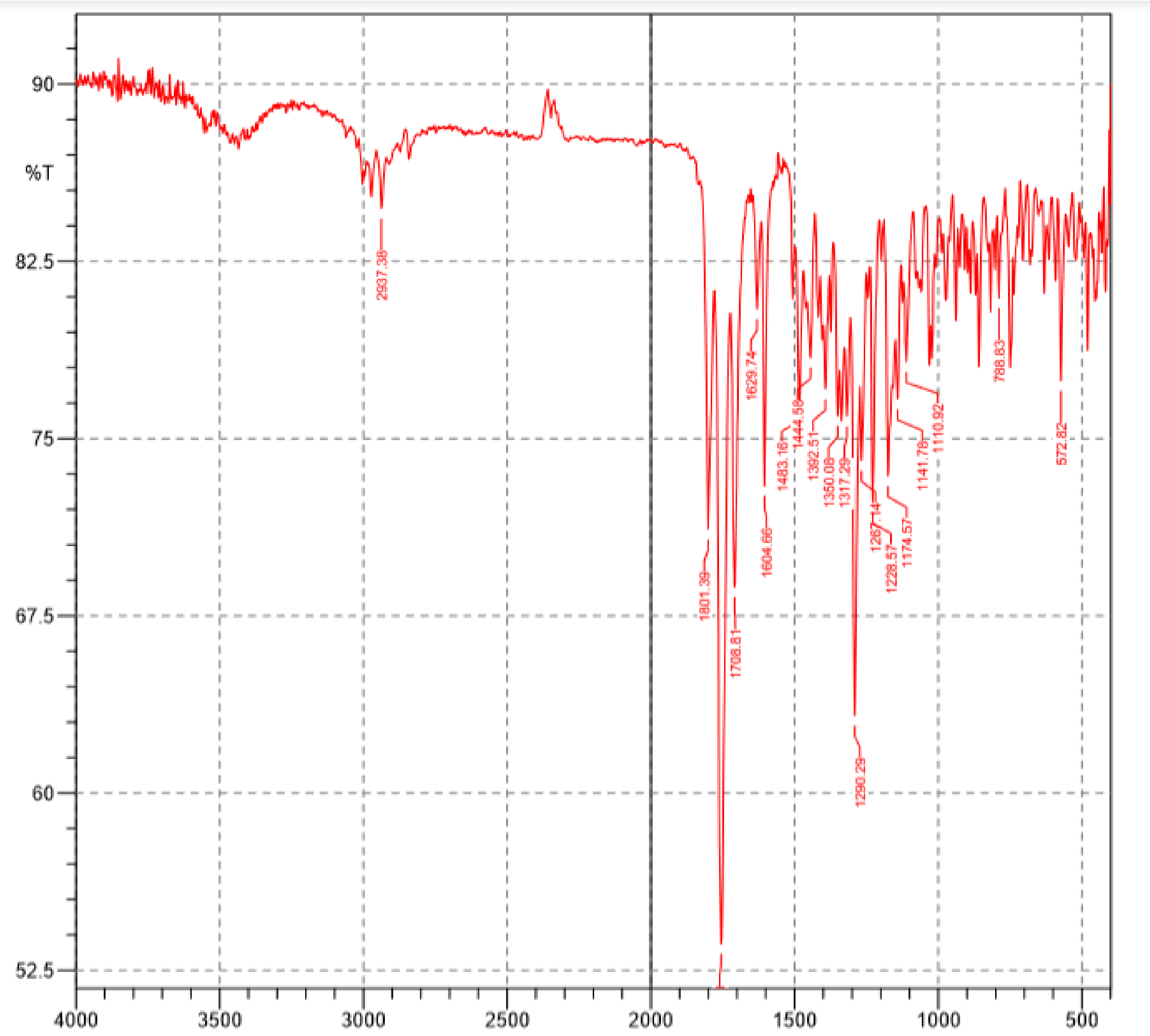

Figure S8 FT-IR spectrum of 1-methylhydantoin-naproxen.

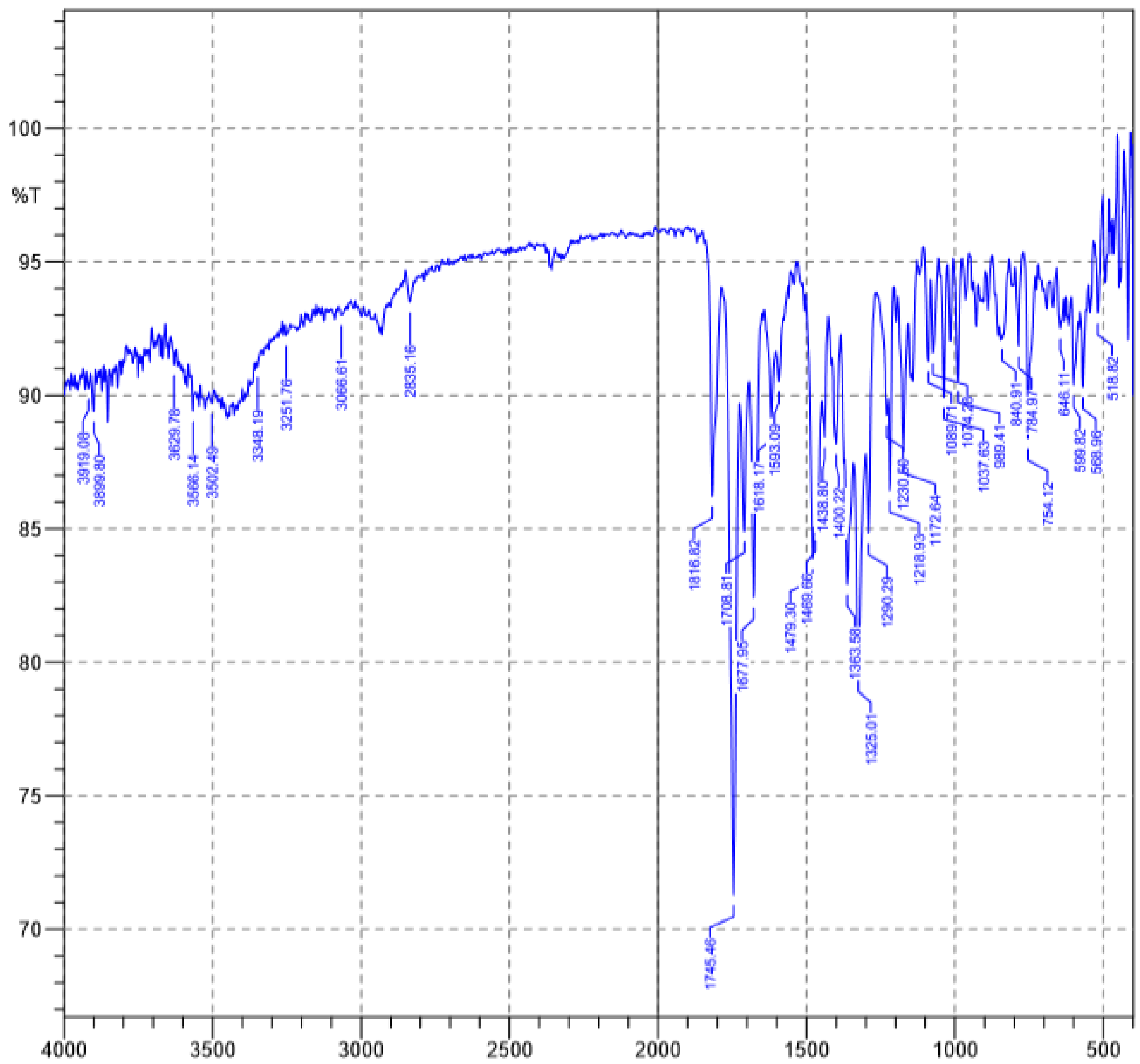

Figure S9 FT-IR spectrum of 1-methylhydantoin-indomethacin.

3. MS Data

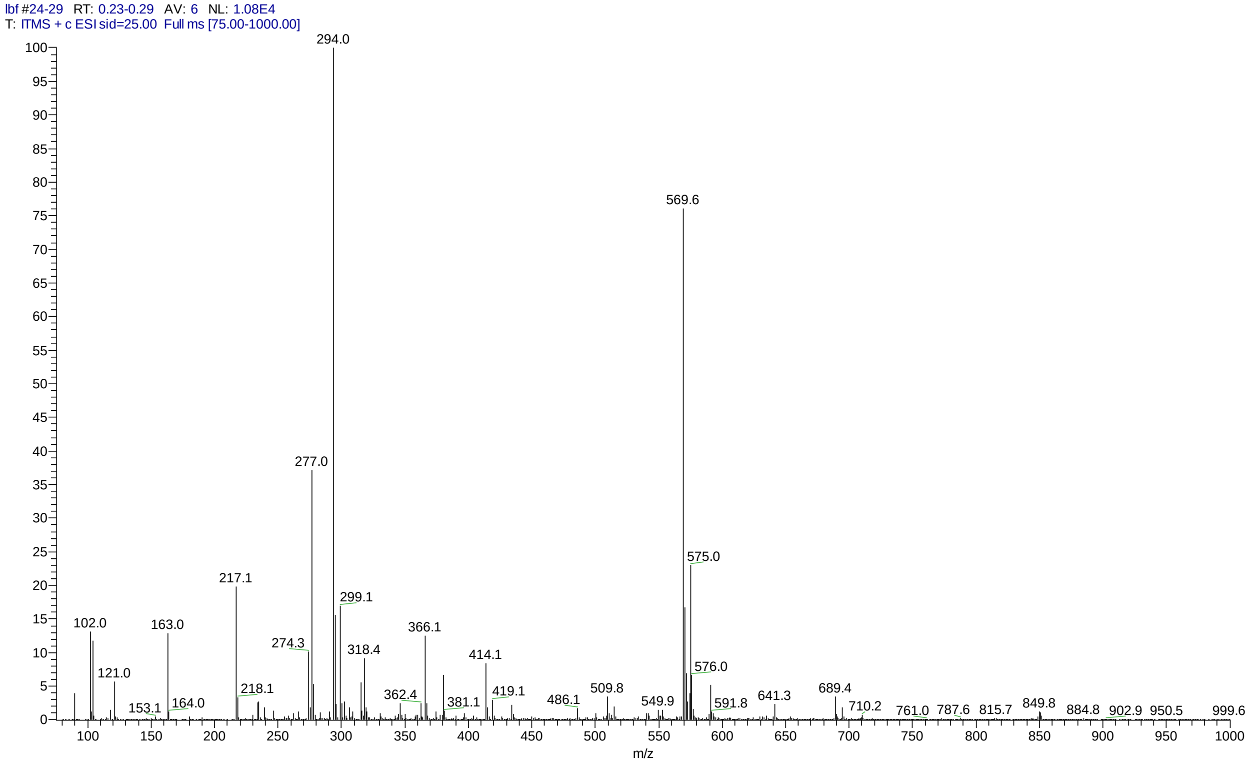

Figure S10 MS result of 1-MHDA.

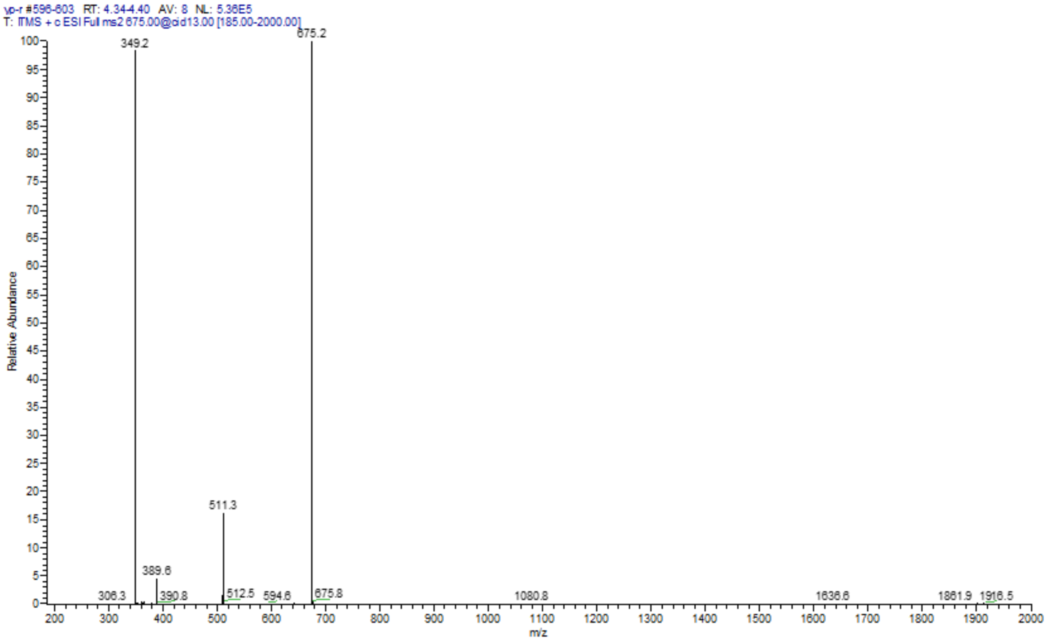

Figure S11 MS spectrum of 1-methylhydantoin-naproxen.

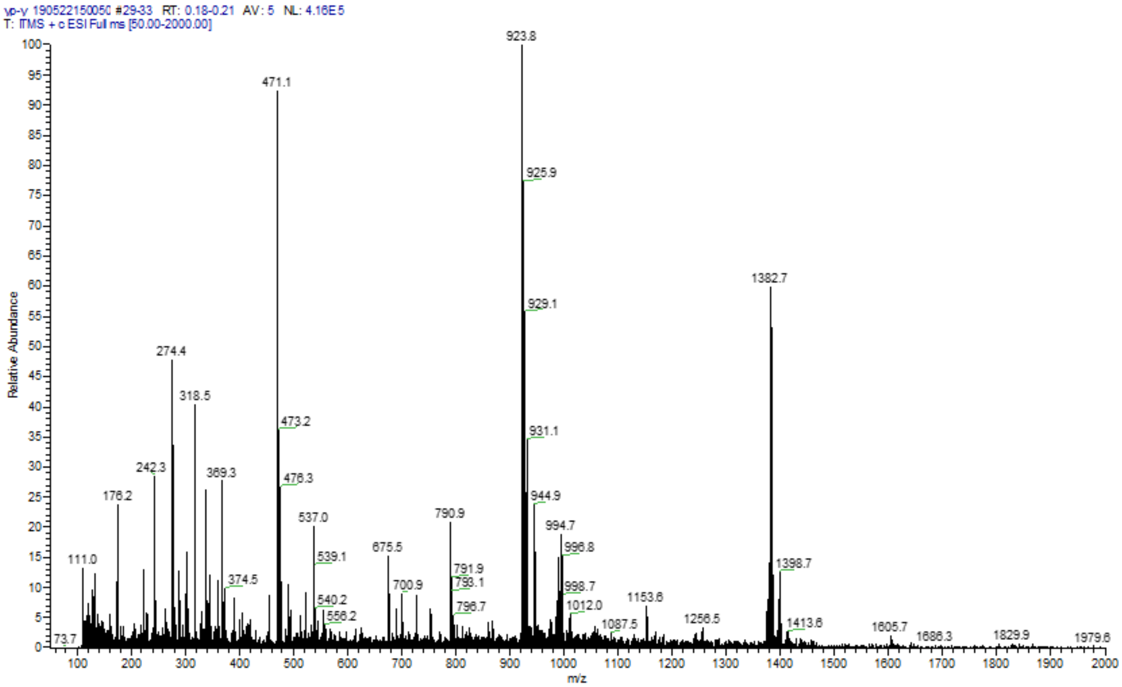

Figure S12 MS spectrum of 1-methylhydantoin-indomethacin.

4. UV/Vis

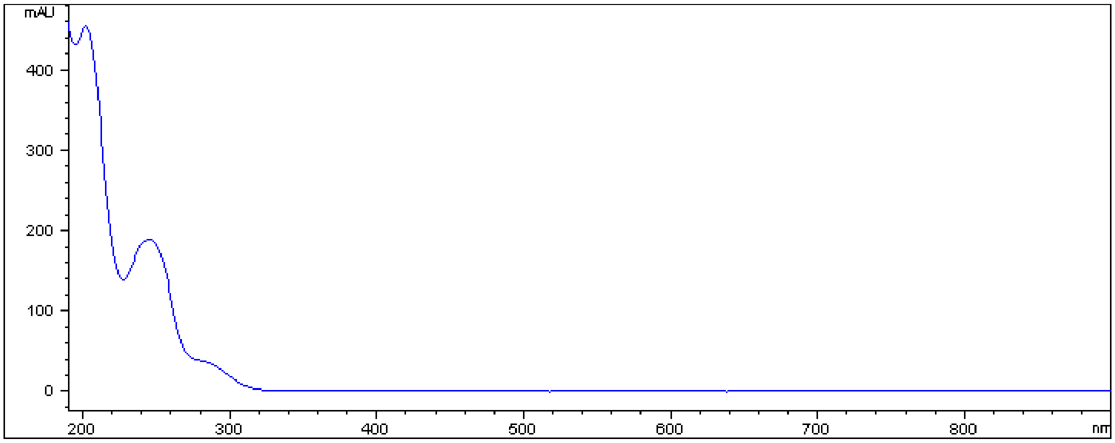

Figure S13 UV/Vis absorbance of 1-MHDA.

5. Crystal Data

Table S1 Crystal data and structure refinement for 1-methylhydantoin-aspirin.

Identification code	1-METHYLHYDANTOIN-ASPIRIN
Empirical formula	$C_{13}H_{12}N_2O_5$
Formula weight	276.25
Temperature/K	191(2)
Crystal system	Monoclinic
Space group	$P2_1/n$
a/Å	9.7923(19)
b/Å	12.148(2)
c/Å	11.535(2)
α/°	90.00
β/°	111.586(3)
γ/°	90.00
Volume/Å^3	1275.9(4)
Z	4
ρ_{calc}g/cm^3	1.438
μ/mm^{-1}	0.112
F(000)	576.0
Crystal size/mm^3	0.21 × 0.19 × 0.11
Radiation	MoKα (λ = 0.71073)
2Θ range for data collection/°	4.68 to 52.78
Reflections collected	7054
Independent reflections	2589 [R_{int} = 0.0280, R_{sigma} = 0.0352]
Data/restraints/parameters	2589/0/183
Goodness-of-fit on F^2	1.115
Final R indexes [I>=2σ (I)]	R_1 = 0.0800, wR_2 = 0.2237
Final R indexes [all data]	R_1 = 0.0922, wR_2 = 0.2332

Table S2 Fractional Atomic Coordinates (×10⁴) and Equivalent Isotropic Displacement Parameters (Å²×10³) for 1-methylhydantoin-aspirin. U_{eq} is defined as 1/3 of of the trace of the orthogonalised U_{IJ} tensor.

Atom	*x*	*y*	*z*	U(eq)
N1	-307(3)	8963(2)	-3498(3)	30.6(6)
N2	-1217(4)	8733(3)	-5562(3)	49.1(9)
O1	3854(3)	8621(2)	902(3)	43.9(7)
O2	1917(2)	9751.4(18)	261(2)	37.7(6)
O3	1975(3)	9307(3)	-2072(3)	53.7(8)
O4	1234(3)	8311(3)	-4478(3)	55.8(8)
O5	-2341(3)	9706(2)	-3221(2)	41.4(7)
C1	4212(4)	10588(4)	1086(4)	47.1(10)
C2	3377(3)	9537(3)	761(3)	31.4(7)
C3	953(3)	8860(2)	-33(3)	30.4(7)
C4	586(4)	8393(3)	901(3)	36.8(8)
C5	-481(4)	7588(3)	608(3)	41.3(9)
C6	-1175(4)	7250(3)	-619(3)	39.8(8)
C7	-812(4)	7722(3)	-1548(3)	32.4(7)
C8	275(3)	8539(3)	-1272(3)	29.4(7)
C9	756(4)	8987(3)	-2253(3)	33.4(8)
C10	-1767(3)	9298(3)	-3876(3)	30.0(7)
C11	-2416(4)	9100(3)	-5218(3)	34.0(8)
C12	-1315(8)	8273(5)	-6722(5)	80.0(17)
C13	51(4)	8627(3)	-4532(4)	36.8(8)

Table S3 Anisotropic Displacement Parameters ($Å^2×10^3$) for 1-methylhydantoin-aspirin. The Anisotropic displacement factor exponent takes the form: $-2\pi^2[h^2a^{*2}U_{11}+2hka^*b^*U_{12}+…]$.

Atom	U_{11}	U_{22}	U_{33}	U_{23}	U_{13}	U_{12}
N1	29.9(14)	29.9(14)	34.3(15)	4.4(11)	14.4(12)	-1.5(11)
N2	74(2)	40.7(18)	43.0(18)	0.5(14)	33.3(18)	1.9(16)
O1	33.8(13)	40.7(15)	58.2(17)	12.2(12)	18.2(12)	8.3(11)
O2	26.8(12)	26.7(12)	53.8(15)	-6.2(11)	8.0(10)	-0.2(9)
O3	29.1(13)	75(2)	52.3(17)	19.8(15)	9.4(12)	-12.9(13)
O4	51.1(17)	55.9(18)	77(2)	-3.9(15)	43.6(16)	1.4(14)
O5	38.3(13)	46.7(15)	37.9(14)	-4.1(11)	12.6(11)	10.3(11)
C1	32.2(19)	49(2)	57(2)	-10.7(18)	13.3(17)	-11.9(16)
C2	27.3(15)	38.0(18)	29.5(16)	-0.4(14)	11.1(13)	-1.1(13)
C3	24.0(15)	22.8(15)	42.7(19)	-2.7(13)	10.3(13)	2.0(12)
C4	39.3(19)	39.1(18)	28.7(16)	-3.9(14)	8.7(14)	3.5(15)
C5	45(2)	47(2)	34.9(18)	6.0(16)	18.1(16)	-3.4(16)
C6	39.1(19)	34.3(18)	47(2)	-2.4(16)	16.5(16)	-12.3(15)
C7	32.6(16)	29.6(16)	33.8(17)	-3.0(13)	10.9(13)	-3.8(13)
C8	24.6(15)	26.2(15)	36.4(17)	2.2(13)	10.2(13)	2.2(12)
C9	27.0(16)	29.1(16)	42.2(19)	5.5(14)	10.4(14)	0.3(13)
C10	29.3(16)	27.7(15)	32.6(17)	-0.2(13)	10.9(13)	-2.2(12)
C11	37.2(18)	29.9(16)	32.0(17)	-2.6(13)	9.4(14)	-4.6(13)
C12	118(5)	65(3)	65(3)	-12(3)	44(3)	-15(3)
C13	45(2)	27.6(16)	46(2)	-1.4(14)	26.4(17)	-5.2(14)

Table S4 Bond Lengths for 1-methylhydantoin-aspirin.

Atom	Atom	Length/Å	Atom	Atom	Length/Å
N1	C10	1.393(4)	O5	C10	1.202(4)
N1	C13	1.421(4)	C1	C2	1.488(5)
N1	C9	1.431(4)	C3	C4	1.378(5)
N2	C13	1.373(5)	C3	C8	1.392(5)
N2	C12	1.420(6)	C4	C5	1.379(5)
N2	C11	1.442(5)	C5	C6	1.388(5)
O1	C2	1.194(4)	C6	C7	1.373(5)
O2	C2	1.355(4)	C7	C8	1.403(4)
O2	C3	1.394(4)	C8	C9	1.480(5)
O3	C9	1.198(4)	C10	C11	1.461(5)
O4	C13	1.199(5)			

Table 5 Bond Angles for 1-methylhydantoin-aspirin.

Atom	Atom	Atom	Angle/°	Atom	Atom	Atom	Angle/°
C10	N1	C13	111.3(3)	C7	C6	C5	120.1(3)
C10	N1	C9	126.3(3)	C6	C7	C8	120.7(3)
C13	N1	C9	122.3(3)	C3	C8	C7	117.8(3)
C13	N2	C12	120.3(4)	C3	C8	C9	121.1(3)
C13	N2	C11	111.1(3)	C7	C8	C9	121.0(3)
C12	N2	C11	127.0(4)	O3	C9	N1	119.5(3)
C2	O2	C3	117.9(2)	O3	C9	C8	124.2(3)
O1	C2	O2	122.4(3)	N1	C9	C8	116.2(3)
O1	C2	C1	127.8(3)	O5	C10	N1	126.0(3)
O2	C2	C1	109.8(3)	O5	C10	C11	127.7(3)
C4	C3	C8	121.7(3)	N1	C10	C11	106.2(3)
C4	C3	O2	118.5(3)	N2	C11	C10	105.2(3)
C8	C3	O2	119.5(3)	O4	C13	N2	128.6(4)
C3	C4	C5	119.5(3)	O4	C13	N1	125.5(4)
C4	C5	C6	120.2(3)	N2	C13	N1	106.0(3)

Table S6 Hydrogen Atom Coordinates (Å×10^4) and Isotropic Displacement Parameters (Å^2×10^3) for 1-methylhydantoin-aspirin.

Atom	*x*	*y*	*z*	U(eq)
H1A	5268	10431	1395	71
H1B	3971	10970	1734	71
H1C	3949	11055	343	71
H4A	1065	8623	1741	44
H5A	-741	7264	1248	50
H6A	-1904	6691	-816	48
H7A	-1301	7493	-2387	39
H11A	-3189	8529	-5412	41
H11B	-2854	9784	-5669	41
H12A	-325	8130	-6710	120
H12B	-1821	8790	-7395	120
H12C	-1866	7581	-6860	120
